# Measuring the motives of informal entrepreneurs

**DOI:** 10.12688/f1000research.73706.2

**Published:** 2022-12-01

**Authors:** Noor Shahaliza Othman, Govindan Marthandan, Kamarulzaman Ab Aziz

**Affiliations:** 1 Faculty of Management, Multimedia University, Cyberjaya, Selangor, 63100, Malaysia

**Keywords:** Necessity driven, Opportunity driven, Motives, Informal entrepreneur

## Abstract

**Background** - Handling non-observed activities pose major challenges to the governments and other stakeholders. Non-observed activities refer to underground activities, illegal activities, informal sector and any other activities that result in goods or services consumed by the household. The impact of these non-observed activities shows that the volume of people involved in the informal sector will rapidly increase. Informal economic activities are technically illegal yet are not intended as antisocial,   thereby remaining acceptable to many individuals within the society. This research aimed to identify the factors that lead to entrepreneurial necessity and opportunity.

**Methods** – The data of 51 respondents who were employed as informal entrepreneurs in Klang Valley areas in Malaysia was collected with the use of a questionnaire and convenient and proportionate sampling techniques. The data were analysed using SPSS software.

**Results **– The two primary drivers of informal entrepreneurial activity were necessity and opportunity. The inability to find a formal job was an example of being driven by necessity. Meanwhile, individuals that are driven by opportunity chose to work independently in these informal sectors. Between necessity and engagement, refinement acted as a mediator. Often, necessity and opportunity do not automatically translate into successful entrepreneurship; further refinement is required in terms of market potential, technology usage, location preferences, and capital requirements. Improved refinement results in increased entrepreneurial engagement.

**Conclusions **- The role and contribution of the informal sector entrepreneurship in economic development need to be evaluated and not just observed as an opportunity for individuals who choose this type of career. Therefore, further research is required in a wider variety of contexts to evaluate whether the same remains true in different populations. The results of this study can be useful for the government to set policies to encourage the transition of informal to formal entrepreneurships in Malaysia.

## Introduction

Non-observed activities typically include underground, illegal, and any other informal sector activities that results in commodities or services that a household can consume. ^1^ As a result of these unregulated activities, the informal sector of the population has increased significantly in recent years. Additionally, despite being legally unlawful, informal economic activities are accepted by a significant portion of society since they are not “antisocial in intent” despite being illegal. ^1^

There has been many competing theories proposed to explain the rise of informal sector entrepreneurship. However, only a handful of people have attempted to document these divergent points of view, and even fewer have attempted an objective assessment of their validity. According to previous research, two major factors significantly impacted entrepreneurs in the informal sector. Push factors, also known as factors of necessity, and pull factors, also known as factors of opportunity. ^2^^–^^4^

According to Williams, C. C., ^3^ push factors can be understood as unemployment, underemployment, and dissatisfaction with current employment. “Necessity” entrepreneurs were forced into entrepreneurship due to a lack of alternatives. These are defined as a desire for independence, self-actualisation, financial benefits, and a desire to achieve a better balance between family and work obligations. Entrepreneurs seize such an “opportunity” because they want to be self-sufficient or own a business. ^2^

Individuals who consider entrepreneurship as the best available option rather than the best option for them personaliy are referred to as necessity refinement types of individuals. ^5^ Opportunity refinement implies to opportunity creation as opportunities usually do not matterialise unexpectedly and therefore must be created. Based on personal “rationale” and “talent”, an individual must decide whether or not to take advantage of the opportunity and select a preferred career path and industry. In actuality, ensuring that entrepreneurs have access to opportunities contributes to economic growth.

The number of informal entrepreneurs in Malaysia is on the rise yearly. ^6^ The hardship and challenges of surviving in the current economic situation compels people to be inclined to engage in types of work that are usually of the informal business type other than their official work.

Like formal and corporate entrepreneurs, informal entrepreneurs foster an informal mode of entrepreneurship in more diverse and creative sectors, which eventually it can significantly contributes to the national economy.

As such, the main objective of this paper is to empirically test the factors that create the need for entrepreneurial necessity and opportunity. The research findings are expected to shed light on why people choose informal entrepreneurship and how they become involved in these types of work over time. As a result, the government will be able to use this information when developing policies on entrepreneurship.

### Informal sector

The informal sector provides substantial subsidies to certain economies, most notably those in developing countries. ^7^ Consequently, this is critical for creating job oppertunities and income generation. For example, in 2005, analyses of the mixed-income of Malaysian households revealed that the informal sector contributed 13% to the country's gross domestic product. ^8^ Although the previous study established that informal sectors are highly competitive andindividualized, the topic of informal sector disputes and competitiveness has had a relatively limited attention in the literature. ^9^^,^^10^

The Organisation for Economic Co-operation and Development (2002) ^11^ and the System of National Accounts (1993) ^12^ have defined the informal sector as “an enterprise that includes households that produce goods solely for personal consumption, thus encompassing all units engaged in productive activities.”

The 15 ^th^ International Conference of Labour Statisticians (1993) ^13^ has also defined the informal sector as:

(a) Characterised. The informal sector can be depicted rather well. These units are frequently associated at a low level, with little or no division in the middle of work and capital as production components, and on a small scale.
(b) The distinctive characteristics of household businesses. The altered and unique possessions utilised do not have a place in the production units, at least not in accordance to their managers. The owners are required to increase the necessary finance at their own risk.

According to the Malaysian Department of Statistics (2012), ^6^ an employed person in the informal sector is defined as a working population in an establishment that meets the following conditions:

(a) The business is not registered with the Malaysian Companies Commission or other professional organisations, including the local government.
(b) All or no less than one product or administration created are implied to be purchased or deal transaction.
(c) The number of people employed is fewer than 10, and the company is not incorporated under a specific type of national legislation.
(d) The establishment is involved in non-agricultural activities.

### Necessity- and opportunity-driven entrepreneurship

While most informal entrepreneurs are found to capitalise on business possibilities, others are formed due to the owner's inability to obtain an adequate job or be obliged to work. When the two factors are examined, it is observed that in Africa, opportunity driven firms are more efficient and widespread than in other parts of the world. ^14^

Originally, Schumpeter's definition of an entrepreneur was someone who is willing to take risks to capitalise on an existing business opportunity and who will start a new business if the idea is good and the opportunity exists. ^15^ However, in developing countries, most businesses are started not because of opportunities but because the owners are unable to find employment in their desired field.

According to the 2009 World Bank Enterprise survey on informal business in Cote d'Ivoire, Madagascar, and Mauritius, 39% of businesses were started by business owners who own the majority of the business but were unable to find job satisfaction. ^16^ The remaining 61% took advantage of the opportunity to start their businesses or expand existing ones. Based on this study, opportunities versus needs regarding business or informal entrepreneurship were related to a business's structure, performance, and problems encountered during operation.

When it comes to running a business, opportunistic entrepreneurs may be more motivated and skilled than other types of entrepreneurs. Nonetheless, entrepreneur must always be prepared to encounter difficulties while conducting business. Typically, informal businesses operate on a small scale and engage in simple business activities.

The widely held belief is that informal sector entrepreneurs in developing countries are primarily necessity-driven individuals forced into entrepreneurship as a means of survival due to the absence of other options. ^5^ Entrepreneurs in the informal sector can benefit from understanding the distinction between opportunity and necessity entrepreneurs. This distinction can be used to analyse acceptable entrepreneurs and better understand the thought processes of entrepreneurs in the informal sector.

Until recently, individuals who work entirely or partially in the informal sector were assumed to be motivated by necessity, pushed into this line of work in the absence of other options.

According to William (2007), ^17^ “Choose to participate in the informal economy because they find more autonomy, flexibility, and freedom in this sector than in the formal one. In other words, participants have the freedom of operating their own business; they have elasticity in defining hours or days of operation; they can use and develop their creativeness.”

Nonetheless, over the last decade or so, the opposite has begun to be discussed. Scholars have begun to refer to them as opportunity entrepreneurs, emphasising the word entrepreneur in the preceding statement. Despite the widespread belief that external pressures (such as economic reform, unemployment, and discrimination) drive people into the informal economy, the majority of the fifty informal sector entrepreneurs has discussed did so voluntarily. ^18^^,^^19^ The majority of chance entrepreneurs came to this sector searching for a new career. Even individuals who began as necessity-driven entrepreneurs are more likely to develop a long-term commitment to their informal sector businesses due to the official economy's limited opportunities.

## Methods

This research adapted positivism discipline as a critical element. This quantitative study involved the collection of data via a structured questionnaire ( Underlying data). ^20^

Questionnaires were specifically prepared by using 5-point Likert-scale: (1) Strongly disagree – (5) Strongly agree, to reflect the agreement of respondents. The questions were also translated into the Malay Language to cater to the responders language preferences. The type of data used was primary data. The unit of analysis was an individual who was an informal entrepreneur. In this cross-sectional study, data was collected personally via face-to-face interview. Joe F. Hair *et al.* ^21^ recommended 30 to 100 for the minimum sample size to provides valid results using SPSS Software (Version 26). Based on the G Power test, the sample size needed was 45 based on effect size and the number of predictors in the research. As a result, it was agreed that 100 questionnaires would be distributed via convenient sampling techniques to individuals who were employed as informal entrepeneurs in Klang Valley areas in Malaysia. This particular area was selected because it had the highest number of employed in the informal sector as reported by the Department of Statistics ^6^ compare to other states. However, only 51 of the 100 questionnaires distributed was used for further analysis. This was because at the screening and cleaning stage, 35 respondents were rejected due toincomplete or missing responses, additionally some respondents had registered businesses, and 16 had operated their businesses for less than a year.

The first section of the questionnaire asked respondents about their history of gross household income, the employment status and employment histories of household members, their ages, gender, and the type of work they relied on the most to maintain their standard of living ( Underlying data). ^20^ The following section contained an open-ended questions about whether they were self-employed or built their business - if they had built their business, they had to answer when these enterprises began, whether they conducted some or all of their transactions in the informal sector and various reasons for starting this venture.

**Figure 1.**
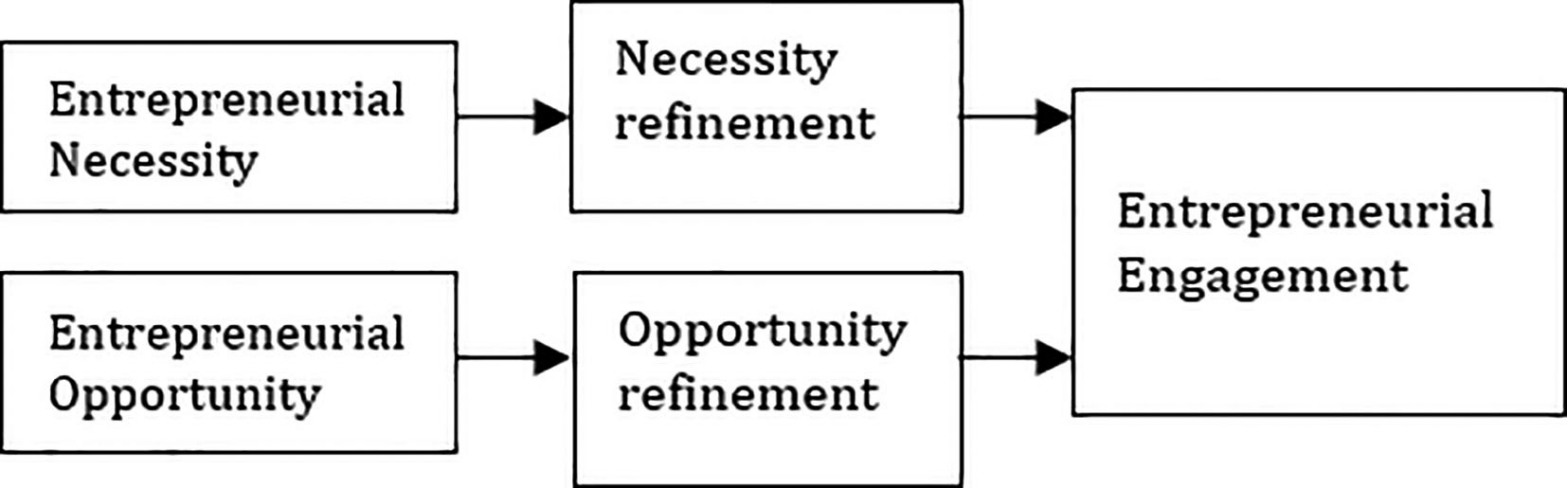
Proposed framework. Adapted from Ref. 20.

Below are the research framework adopted for this study ( Figure 1).

### Ethical statement

Prior to conducting this study ethical approval was obtained from the Research Ethical Committee (Approval Number: EA2542021) of Multimedia University. Participants provided informed oral consent to participate in the study. Oral consent instead of written consent was obtained because of time constraints and illiteracy of some participants.

## Results

### Characteristics of respondents

According to this survey, 53% of the respondents are male and 47% are female. The characteristics of respondents is shown in Table 1.

**Table 1.** Characteristics of respondents.

		Percentage
**Gender**	Male	53%
	Female	47%
**Age**	19–24	12%
	25–39	65%
	40–59	23%
**Status**	Single	14%
	Married	73%
**Monthly income (RM)**	500–1000	41%
	1001–1500	27%
	>1501	32%
**Education**	High school	53%
	Diploma	20%
	Degree and above	27%

According to the results, 65% of respondents were married between the ages of 25 and 39. The committed married couples had mentioned that in order to provide for their families, they will take on any task. On the other hand, 41% of them earned less than RM1000 per month and had only completed secondary school Based on these findings education has a strong correlation with income. Therefore, it is critical to acquire a high degree of education to earn a higher salary. According to the demographic profile of the respondents, their competence and productiveness were in the medium range, which is consistent with previous studies. The vast majority of them conducted their business in person with the customers.They interacted directly with customers, and some operateed their businesses from their homes.

### Motives of informal entrepreneurship

According to Table 2, 53% of respondents identified themselves as “employed” informal entrepreneurs due to the opportunity-driven environment. From this list of opportunity-driven alternatives, most respondents (48%) choose to be informal entrepreneurs due to the time flexibility and preference for self-employment. That was the primary reason for setting up a business. Additionally, making time for other commitments such as family commitments, was important. Thus, time flexibility was one of the most critical criteria for informal entrepreneurs.

**Table 2.** Motives of informal entrepreneurs.

Necessity-driven	47%	Opportunity-driven	53%
Did not find a formal job	4%	Independence	15%
Extra income	21%	Experience in business	11%
Dissatisfaction with previous employment	33%	Family tradition	19%
Help family/spouse	42%	Time flexibility	48%
		Secondary job	7%

Another factor was family tradition, which accounted for 19% and past business experience, which accounted for 11% of the votes. However, necessity-driven factors such as the need to earn additional income to support their family, dissatisfaction with previous employment, the need to supplement net income, and an inability to find suitable jobs were cited as reasons for starting their businesses by 47% of informal entrepreneurs.

## Discussion

As the informal entrepreneurial process can add value to socio-economic status of Malaysia through growth of the household income, welfare, confidence development, introduction of social and political peace, and improvement in income distribution and demographic phenomenon, an in-depth investigation due to its uniqueness. The findings of this study would be able to assist the Malaysian government in understanding the needs and desires of entrepreneurs, especially in informal entrepreneurship that is lacking and deficient in terms of profound knowledge and understanding of the development and sustainability of informal entrepreneurship with all its strengths and complex processes. Additionally, education is critical in guiding informal entrepreneurs. As a result, informal sector entrepreneurship should be reinterpreted as typically driven by opportunity, however its role in economic development should be examined. Additional studies are needed to challenge the image of entrepreneurship in the informal sector by assessing factors such as the level of education or political stance in certain communities, the rigidness of transitioning from informal to formal business, and also the policies available to harness this large area of entrepreneurship on a global scale.

## Conclusions

As this is a cross-sectional study, the data cannot be utilised to establish a solid causal relationship based on the study's findings. As a result, a longitudinal studies are needed to examine the correlations between the study variables. In this study, which was prone to methodological bias, self-report data were utilised to create connections between variables. To minimise bias in future studies, closed envelopes should be used to guarantee confidentiality and anonymity throughout the data gathering process. Some respondents may underreport their informal entrepreneurial motivations out of concern for the implications of future legal actions on their business. This could result in reporting bias, which would have an effect on the study's results. To mitigate this, each study participant should be informed clearly about the study's goal and ensured that the information would be kept anonymous.

## Data availability

### Underlying data

Figshare: Measuring the motives of informal entrepreneurs https://figshare.com/s/c70b053eacb5ac8a24e4. ^20^

This project contains the following underlying data:

Data file 1. Questionnaire https://figshare.com/s/b9433876684c7fd89572. ^22^

This project contains the following underlying data: Data file 1. MiniFund Questionnaire Report

Data are available under the terms of the Creative Commons Zero “No rights reserved” data waiver Attribution 4.0 International (CC BY 4.0).

## Author contributions

Noor Shahaliza Othman, Govindan Marthandan and Kamarulzaman Ab Aziz were involved in overall direction and planning and supervised the work. Noor Shahaliza developed the research framework, carried out the implementation and analysed the data. Govindan Marthandan and Kamarulzaman Ab Aziz advised on the execution process including supervised the progress.

## References

[1] Webb JW Bruton GD Tihanyi L : Research on entrepreneurship in the informal economy: Framing a research agenda. *J. Bus. Ventur.* 2013;28:598–614. 10.1016/j.jbusvent.2012.05.003

[2] Africa S Africa S : Retheorising the Motives of Informal Ent. 2007.

[3] Williams CC : Beyond necessity-driven versus. 2008;9(3):1–10.

[4] Bora RS : Migrant Informal Workers: A Study of Delhi and Satellite Towns. *Mod. Econ.* 2014;05(05):562–579. 10.4236/me.2014.55053

[5] Williams CC Youssef Y : Is Informal Sector Entrepreneurship Necessity- or Opportunity-driven? Some Lessons from Urban Brazil. 2014;3(1):41–53. 10.5430/bmr.v3n1p41

[6] Department of Statistics: *Informal sector workforce survey report.* Kuala Lumpur: Department of Statistics, Malaysia;2012.

[7] Gerxhani K : The informal sector in developed and less developed countries: a literature survey. *Public Choice.* 2004;120(3):267–300. 10.1023/B:PUCH.0000044287.88147.5e

[8] Baharudin N Othman M Waty P : Informal Employment in Informal Sector Enterprises In Malaysia Abstrak. Jabatan Statistik Malaysia. 2006.

[9] Beyer A : Motivations for Engaging in Entrepreneurial Activity in the Informal Sector in Sub Saharan Africa. 2018.

[10] Brouwer S Krol B Reneman MF : Behavioral Determinants as Predictors of Return to Work after Longterm Sickness Absence: An Application of the Theory of Planned Behavior. *J. Occup. Rehabil.* 2009;19(2):166–174. 10.1007/s10926-009-9172-5 19333738

[11] Organisation for Economic Co-operation and Development Staff: *Education at a glance: OECD indicators 2002.* Paris: OECD;2002.

[12] Inter-Secretariat Working Group on National Accounts:Brussels/Luxembourg, New York, Paris, Washington, D.C., Pok, C. a frontera de la 1993.1993.

[13] Hussmanns R : Statistical definition of informal employment: Guidelines endorsed by the Seventeenth International Conference of Labour Statisticians (2003). *7th Meeting of the Expert Group on Informal Sector Statistics (Delhi Group).* 2004, February; (pp.2–4).

[14] Amin M : Necessity vs. Opportunity Entrepreneurs in the Informal Sector. 2008.

[15] Schumpeter JA : Entrepreneurship as innovation. *University of Illinois at Urbana-Champaign's Academy for Entrepreneurial Leadership Historical Research Reference in Entrepreneurship.* 2000.

[16] Williams CC Kedir AM : Explaining cross-country variations in the prevalence of informal sector competitors: lessons from the World Bank Enterprise Survey. *Int. Entrep. Manag. J.* 2019;15(3):677–696. 10.1007/s11365-018-0527-2

[17] Williams CC : Entrepreneurs operating in the informal economy: necessity or opportunity driven? *J. Small Bus. Entrep.* 2007;20(3):309–319. 10.1080/08276331.2007.10593402

[18] Snyder JM Yackovlev I : Political and Economic Determinants of Changes in Government Spending on Social Protection Programs. 2000.

[19] Al-Mataani R Wainwright T Demirel P : Hidden Entrepreneurs: Informal Practices within the Formal Economy. *Eur. Manag. Rev.* 2017;14(4):361–376. 10.1111/emre.12115

[20] Othman NS Marthandan G Aziz KA : Measuring the motives of informal entrepreneurs. 2021. Reference Source

[21] Hair JF Matthews LM Matthews RL : PLS-SEM or CB-SEM: Updated Guidelines on Which Method to Use. *International Journal Multivariate Data Analysis.* 2017;1(2):107–123. 10.1504/IJMDA.2017.087624

[22] Othman NS Marthandan G Aziz KA : Measuring the motives of informal entrepreneurs. 2021. Reference Source

