# Supplementary material for: Measuring the motives of informal entrepreneurs
Source: F1000Res. 2022 Dec 1;11:56. Originally published 2022 Jan 18. [Version 2] doi: 10.12688/f1000research.73706.2 (PMC9745207; doi:10.12688/f1000research.73706.2)
Supplement: Peer review history

# Peer review: Reviewer response for version 2

**DOI:** 10.5256/f1000research.136299.r157107

**Published:** 2022-12-12

**Authors:** Muhammad Ashraf Fauzi

**Affiliations:** 1 Faculty of Industrial Management, Universiti Malaysia Pahang, Kuantan, Malaysia

Accepted

Is the work clearly and accurately presented and does it cite the current literature?

Yes

If applicable, is the statistical analysis and its interpretation appropriate?

Yes

Are all the source data underlying the results available to ensure full reproducibility?

Yes

Is the study design appropriate and is the work technically sound?

Yes

Are the conclusions drawn adequately supported by the results?

Yes

Are sufficient details of methods and analysis provided to allow replication by others?

Yes

Reviewer Expertise:

Knowledge Management, Entrepreneurship

I confirm that I have read this submission and believe that I have an appropriate level of expertise to confirm that it is of an acceptable scientific standard.

# Peer review: Reviewer response for version 2

**DOI:** 10.5256/f1000research.136299.r157106

**Published:** 2022-12-02

**Authors:** Gurel Cetin

**Affiliations:** 1 Tourism Management Department, Faculty of Economics, Istanbul University, İstanbul, Turkey

The authors addressed my comments.

Is the work clearly and accurately presented and does it cite the current literature?

No

If applicable, is the statistical analysis and its interpretation appropriate?

Partly

Are all the source data underlying the results available to ensure full reproducibility?

Yes

Is the study design appropriate and is the work technically sound?

No

Are the conclusions drawn adequately supported by the results?

No

Are sufficient details of methods and analysis provided to allow replication by others?

Yes

Reviewer Expertise:

entrpreneuship

I confirm that I have read this submission and believe that I have an appropriate level of expertise to confirm that it is of an acceptable scientific standard.

# Peer review: Reviewer response for version 1

**DOI:** 10.5256/f1000research.77375.r125700

**Published:** 2022-03-04

**Authors:** Muhammad Ashraf Fauzi

**Affiliations:** 1 Faculty of Industrial Management, Universiti Malaysia Pahang, Kuantan, Malaysia

Thank you for providing the opportunity to review this work. These are my comments:

1. The last sentence in the first para "In addition, while informal economic activities are technically illegal, they are not “antisocial in intent,” making them acceptable to a large segment of society despite being illegal."
  - This sentence requires more explanation as it seems hanging. What does society correspond to it being illegal?
2. Some of the paragraphs are too short. Example "Like formal and corporate entrepreneurs, informal entrepreneurs foster an informal mode of entrepreneurship in more diverse and creative sectors, which eventually it can significantly contributes to the national economy"
  - This paragraph should be combined with the previous.
3. There are some spelling mistakes "onvolved" for example in page 4. Another is "thisstudy".
4. The discussion is not enough. Please provide evidence and support from past studies that are in congruence with the author's findings.
5. The conclusion is actually a limitation, please reiterate and conclude why informal entrepreneurship is important to the society/nation-building, etc.
6. The manuscript requires proofreading, as there are grammar mistakes, spelling and other minor errors.

Is the work clearly and accurately presented and does it cite the current literature?

Yes

If applicable, is the statistical analysis and its interpretation appropriate?

Yes

Are all the source data underlying the results available to ensure full reproducibility?

Yes

Is the study design appropriate and is the work technically sound?

Yes

Are the conclusions drawn adequately supported by the results?

Yes

Are sufficient details of methods and analysis provided to allow replication by others?

Yes

Reviewer Expertise:

Knowledge Management, Entrepreneurship

I confirm that I have read this submission and believe that I have an appropriate level of expertise to confirm that it is of an acceptable scientific standard, however I have significant reservations, as outlined above.

# Peer review: Reviewer response for version 1

**DOI:** 10.5256/f1000research.77375.r120473

**Published:** 2022-02-17

**Authors:** Gurel Cetin

**Affiliations:** 1 Tourism Management Department, Faculty of Economics, Istanbul University, İstanbul, Turkey

1. The paper looks into an important area of informal entrepreneurship, but does not explain how are they different t formal entrepreneurs. Moreover, "between necessity and engagement, refinement acted as a mediator" - there is no finding or analysis concerning refinement as a mediator. Please explain.
2. The sample is too small, particularly for a SEM study. The authors tried to justify this but it is not really acceptable even if a normal distribution is assumed. King valley should be justified. Why it has been selected? How representative is it? Why not other sites?
3. The literature is somewhat weak concerning the theoretical background. Push and pull factors of entrepreneurship are also described in "Alrawadieh, Z., et al. (2021). The interface between hospitality and tourism entrepreneurship, integration and well-being: A study of refugee entrepreneurs." Please refer to this paper. 
4. The scales and measures used in the study should be described and justified.
5. "The following section contained open-ended questions about whether they were self-employed or built their business" - These are not really exclusive since someone can be both self-employed and build their business.
6. The discussion and conclusions should be elaborated to include theoretical and practical contributions.

Is the work clearly and accurately presented and does it cite the current literature?

No

If applicable, is the statistical analysis and its interpretation appropriate?

Partly

Are all the source data underlying the results available to ensure full reproducibility?

Yes

Is the study design appropriate and is the work technically sound?

No

Are the conclusions drawn adequately supported by the results?

No

Are sufficient details of methods and analysis provided to allow replication by others?

Yes

Reviewer Expertise:

entrpreneuship

I confirm that I have read this submission and believe that I have an appropriate level of expertise to state that I do not consider it to be of an acceptable scientific standard, for reasons outlined above.

## References

[1] : The interface between hospitality and tourism entrepreneurship, integration and well-being: A study of refugee entrepreneurs. *International Journal of Hospitality Management* .2021;97: 10.1016/j.ijhm.2021.103013 10.1016/j.ijhm.2021.103013
